# Encapsulation of Andrographolide in poly(lactide-co-glycolide) Nanoparticles: Formulation Optimization and *in vitro* Efficacy Studies

**DOI:** 10.3389/fbioe.2021.639409

**Published:** 2021-02-18

**Authors:** Bukola A. Oseni, Chukwuemeka P. Azubuike, Omotunde O. Okubanjo, Cecilia I. Igwilo, Jayanth Panyam

**Affiliations:** ^1^Department of Pharmaceutics and Pharmaceutical Technology, University of Lagos, Lagos, Nigeria; ^2^Department of Pharmaceutics, University of Minnesota, Minneapolis, MN, United States; ^3^School of Pharmacy, Temple University, Philadelphia, PA, United States

**Keywords:** andrographolide, poly(lactide-co-glycolide), nanoparticles, formulation optimization, breast cancer

## Abstract

Andrographolide is a potential chemopreventive and chemotherapeutic agent that suffers from poor aqueous solubility. Encapsulation in poly(lactide-co-glycolide) (PLGA) nanoparticles can overcome solubility issues and enable sustained release of the drug, resulting in improved therapeutic efficacy. In this study, andrographolide was encapsulated in PLGA nanoparticles via emulsion solvent evaporation technique. Effect of various formulation parameters including polymer composition, polymer molecular weight, polymer to drug ratio, surfactant concentration and the organic solvent used on nanoparticle properties were investigated. A selected formulation was used to determine the effect of encapsulation in nanoparticles on andrographolide’s *in vitro* anticancer efficacy. Nanoparticles formulated using a polymer with 85:15 lactide to glycolide ratio and ethyl acetate as the organic solvent were found to be optimal based on average hydrodynamic particle size (135 ± 4 nm) and drug loading (2.6 ± 0.6%w/w). This formulation demonstrated sustained release of andrographolide over 48 h and demonstrated significantly greater *in vitro* anticancer efficacy compared to free drug in a metastatic breast cancer cell line. These results suggest that additional, more in-depth efficacy studies are warranted for the nanoparticle formulation of andrographolide.

## Introduction

According to GLOBOCAN—a cancer database created by the International Agency for Research on Cancer (IARC), there is an increase in the global incidence and deaths due to cancer. In 2012, approximately 14.1 million new cases and deaths were recorded (Ferlay et al., 2015) rising to about 18.1 million new incidence and 9.6 million deaths in 2018 (Bray et al., 2018; Ferlay et al., 2019). Breast cancer remains the most common cancer type globally as well as the cause of most deaths associated with cancer in women. In 2018, breast cancer accounted for 24.2% incidence and 15.0% cancer-related deaths in women (Bray et al., 2018; Ferlay et al., 2019).

Chemotherapy is a mainstay treatment modality employed in the management of cancer. Chemotherapeutic agents such as doxorubicin, cisplatin, and paclitaxel have been utilized in the treatment of breast cancer (Takimoto and Calvo, 2005); however, the major drawback includes the development of resistance and life-threatening side effects (due to non-specificity of the chemical agents to cancerous cells) such as cardiac toxicity, hair loss, bone marrow suppression, and gastrointestinal tract lesions, amongst others (Igarashi, 2008; Monsuez et al., 2010; Nussbaumer et al., 2011). An ideal chemotherapeutic agent will exhibit minimal or no side effects while having intrinsic ability to prevent the development of resistance. No such drug currently exists. Research into new therapeutic agents with the aim of overcoming the above limitations therefore continues to be highly relevant. Natural products of plant origin present a source of potential drug molecules (Hosseini and Ghorbani, 2015); many phytochemicals have been shown experimentally to possess cytotoxic activity against various cancer types (Hadjzadeh et al., 2006; Shu et al., 2010; Tan et al., 2011; Wilken et al., 2011; Lè Ne Teiten et al., 2013).

Andrographolide is a labdane diterpenoid derived from the *Andrographis paniculata* plant (Niranjan et al., 2010; Jayakumar et al., 2013). It is the major bioactive compound in the plant and has been found to possess antimicrobial, hepatoprotective, anticancer, anti-inflammatory, and immunostimulatory activities (Jarukamjorn and Nemoto, 2008; Levita et al., 2010; Lim et al., 2012). The cytotoxic activity of this molecule against various cancer types including ovarian, lung, hepatoma, breast, prostate, and colon cancer has been attributed to its ability to act on several cell signaling pathways. Andrographolide exerts direct chemotherapeutic activity via cell cycle arrest at the G0/G1 or G2/M phase. In addition, the drug has also been shown to induce increased production of interleukin 2 (IL-2) and interferon gamma (IFN-γ), which activate cytotoxic T lymphocytes as well as TNF related apoptosis inducing ligand (TRAIL) and death receptors, which eventually leads to apoptosis (Ajaya Kumar et al., 2004; Sheeja and Kuttan, 2007; Mishra, 2016). In addition, the drug inhibits the generation of pro-inflammatory mediators such as tumor necrosis factor alpha (TNF alpha) and angiogenesis mediators such as vascular endothelial growth factor (VEGF) and nitric oxide (NO). In order to combat cancer resistance, it has been suggested that drug molecule(s) activating different death pathways should be utilized. Therefore, a drug molecule such as andrographolide having multiple mechanisms of anticancer activity will be a suitable candidate. Andrographolide, however, has low aqueous solubility, poor bioavailability, and short half-life, resulting in reduced therapeutic activity (Roy et al., 2012; Ghosh et al., 2016). These barriers can be mitigated by the use of a suitable delivery system. In our previous study, andrographolide was formulated into an emulsion, with the particle size in the micrometer range (Oseni et al., 2020). However, microparticles have relatively low cell uptake and poor tissue penetration. Particles in the nanometer size range (nanoparticles) are more advantageous because of their ability to passively accumulate in tumors via the “enhanced permeability and retention effect” (Bharathala and Sharma, 2019).

Nanoparticles are typically fabricated using natural or synthetic polymers such as chitosan, gelatin, albumin, poly(lactide-co-glycolide) (PLGA), polylactide (PLA), and hyaluronan, amongst others (Pal et al., 2011; Bhatia, 2016). They have been used as carriers for the delivery of small molecules, biologic macromolecules, diagnostic agents, and vaccines (Petros and DeSimone, 2010; Bahrami et al., 2017). Encapsulation in nanoparticles can overcome poor aqueous solubility issues because appropriately formulated nanoparticles exhibit excellent suspension stability in biologic fluids (Jacob et al., 2020). PLGA is an FDA-approved synthetic polymer widely used to formulate drug carriers because the polymer is biocompatible and biodegradable, and nanoparticles formulated using PLGA allow for sustained release of various types of payload and can be surface functionalized for targeted delivery applications (Kumari et al., 2010; Danhier et al., 2012; Gentile et al., 2014; Rizvi and Saleh, 2018).

In this study, the encapsulation of andrographolide in PLGA nanoparticles was explored, and the effect of various formulation parameters such as organic solvent, molecular weight of polymer, and lactide:glycolide ratio on key nanoparticle properties such as size, drug loading (DL), and drug release rate were characterized. The formulation of choice with desired physical properties, optimum DL, and *in vitro* release profile was subjected to *in vitro* cytotoxicity studies using LM2 breast cancer cells (a metastatic variant of the MDA-MB-231 triple negative breast cancer parent cells). Our study shows that the optimized nanoparticulate formulation of andrographolide demonstrates greater and more sustained cytotoxic effect vis à vis the free drug.

## Materials and Methods

### Materials

Poly(lactide-co-glycolide) polymer of various lactide:glycolide ratios (50:50, 65:35, 75:25, 85:15, 100:0) as well as of different molecular weights (0.26–0.54, 0.55–0.75, 0.76–0.94, and 0.95–1.20 dL/g inherent viscosity; all of them were 50:50 lactide to glycolide ratio) was purchased from LACTEL (Birmingham AL). Polyvinyl alcohol (87–90% hydrolyzed, MW 30,000–70,000 Da; PVA), flow buffer, RNase, propidium iodide, dimethylsulfoxide (DMSO), phenazine methosulfate (PMS), and andrographolide were purchased from Sigma Aldrich (St. Louis, MO, United States); tween 20 and all organic solvents (HPLC grade) were purchased from Fischer Scientific (Rockford, IL, United States); phosphate buffered saline (PBS), minimum essential medium (MEM), fetal bovine serum (FBS), penicillin, and streptomycin were procured from Gibco; MTS [3-(4,5-dimethylthiazol-2-yl)-5-(3-carboxymethoxyphenyl)-2-(4-sulfophenyl)-2H-tetrazolium] reagent was obtained from Promega; and 0.5%w/v uranyl acetate was procured from VWR (Radnor PA).

#### Cell Line

The LM2 breast cancer cell line was cultured in MEM supplemented with 10% FBS, 100 UI/mL of penicillin, and 100 μg/mL streptomycin (referred to as complete media).

### Methods

#### Preparation of Andrographolide Nanoparticles

The andrographolide-loaded PLGA nanoparticles were prepared using the emulsion solvent evaporation method (Toti et al., 2011; Kim et al., 2018a). Briefly, PVA was dispersed in DI water to obtain the aqueous phase; andrographolide and PLGA were dissolved in 1 mL of chloroform and 200 μL methanol. The organic phase was added into 8 mL of the PVA solution. The mixture was sonicated at 18–20 W for 5 min over ice bath using a probe sonicator (Sonicator XL, Misonix, Melville, NY). The resulting emulsion was placed on the magnetic stirrer (Super-Nuova, Swedesboro, NJ) for 17 h to remove the organic solvent. The nanoparticle suspension formed was then placed under vacuum for 1 h to remove residual organic solvent. The nanoparticles were recovered via centrifugation (Optima XPN-80 Ultracentrifuge, Beckman Coulter Inc., Fullerton, CA) at 35,000 rpm for 35 min and washed three times with DI water and recovered by ultracentrifugation between each washing step. After the final wash step, nanoparticles were resuspended in DI water, lyophilized (Labconco FreeZone 4.5, Kansas City, MO), and stored at −20°C till further analysis.

The effect of formulation variables (lactide:glycolide ratio, inherent viscosity of polymer, organic solvent, surfactant concentration, and drug to polymer ratio) was evaluated for optimization of the formulation (Table 1). Some minor modifications were made in the formulation fabricated using ethyl acetate as the organic solvent because of the lower solubility of the polymer in the solvent. Briefly, andrographolide and PLGA were dissolved in 1.7 mL of ethyl acetate and 330 μL of methanol. The organic phase was added to 8 mL of the aqueous phase. The mixture was sonicated at 18–20 W for 5 min using a probe sonicator, and the emulsion was placed on a magnetic stirrer for 17 h in ambient conditions and further under vacuum for 1 h. Nanoparticles were recovered via centrifugation at 45,000 rpm for 1 h and washed three times with DI water. The nanoparticles were resuspended in DI water, lyophilized, and stored at −20°C for further analysis.

**TABLE 1:** Constituents of the formulation showing the parameters investigated to obtain the optimized formulation.

**Formulation code**	**Lactide to glycolide ratio**	**Inherent viscosity (dL/g)**	**Organic solvent**	**Drug to polymer ratio**	**PVA surfactant concentration**
**Lactide:glycolide**					
A1^a^	50:50	0.55–0.75	Chloroform	1:6	2.5
A2	65:35	0.75	Chloroform	1:6	2.5
A3	75:25	0.55–0.75	Chloroform	1:6	2.5
A4	85:15	0.64	Chloroform	1:6	2.5
A5	100:0	0.55–0.75	Chloroform	1:6	2.5
**Organic solvent**					
B1^a^	50:50	0.55–0.75	Chloroform	1:6	2.5
B2	50:50	0.55–0.75	Dichloromethane	1:6	2.5
B3^b^	50:50	0.55–0.75	Ethyl acetate	1:6	2.5
B4	50:50	0.55–0.75	Acetone	1:6	2.5
**Inherent viscosity**					
C1	50:50	0.26–0.54	Ethyl acetate	1:6	2.5
C2^b^	50:50	0.55–0.75	Ethyl acetate	1:6	2.5
C3	50:50	0.76–0.94	Ethyl acetate	1:6	2.5
C4	50:50	0.95–1.20	Ethyl acetate	1:6	2.5
**Drug:Polymer**					
D1	50:50	0.55–0.75	Ethyl acetate	1:20	2.5
D2	50:50	0.55–0.75	Ethyl acetate	1: 12	2.5
D3^c^	50:50	0.55–0.75	Ethyl acetate	1:8.5	2.5
D4^b^	50:50	0.55–0.75	Ethyl acetate	1:6	2.5
D5	50:50	0.55–0.75	Ethyl acetate	1:4	2.5
**Surfactant concentration**					
E1	50:50	0.55–0.75	Ethyl acetate	1:8.5	1
E2	50:50	0.55–0.75	Ethyl acetate	1:8.5	2
E3^c^	50:50	0.55–0.75	Ethyl acetate	1:8.5	2.5
E4	50:50	0.55–0.75	Ethyl acetate	1:8.5	3
E5	50:50	0.55–0.75	Ethyl acetate	1:8.5	4
F*	85:15	0.64	Ethyl acetate	1:8.5	2

a, b, and c, same formulation variables; F*, optimized formulation.

#### Characterization of Andrographolide PLGA Nanoparticles

##### Particle size, polydispersity index, and zeta potential

The hydrodynamic diameter and polydispersity index (PI) of nanoparticles were determined via dynamic light scattering (DLS) technique (Delsa^TM^ Nano C, Beckman Coulter, Inc.) (Kim et al., 2018a,b). The zeta potential was determined by measuring the electrophoretic mobility of the particles using Delsa^TM^ Nano C. Nanoparticle suspension in DI water was sonicated for 30 s prior to analyses.

##### Surface morphology

Morphology of nanoparticles was determined using transmission electron microscopy (TEM) (FEI Tecnai G2 F30) (Grabowski et al., 2015). Nanoparticle suspension (1 mg/mL) in DI water was deposited on a copper grid. A 0.5% w/v uranyl acetate solution was added as negative stain, excess suspension was blotted out using a filter paper, and the grid was air dried and thereafter observed under the electron microscope.

##### Drug loading and encapsulation efficiency

Standard concentrations of 5–30 μg/mL in methanol of andrographolide reference standard were prepared and placed in a quartz cuvette; the absorbance of the various andrographolide solution prepared was obtained using an ultraviolet (UV) spectrophotometer (Beckman Coulter, Inc.) at 224 nm wavelength. A graph of absorbance against concentration of andrographolide was plotted. The UV method was validated in line with the International Conference on Harmonization guideline (International Conference on Harmonization, 2005).

Andrographolide was extracted from nanoparticles using methanol (1 mg/mL, 1 mL); the methanol was added to the nanoparticles and placed on a rotating shaker for 18 h (Toti et al., 2011). The dispersion was centrifuged at 13,000 rpm for 20 min, dilution of the supernatant was carried out, and the absorbance of the resulting solution was obtained at 224 nm wavelength. The procedure was repeated for nanoparticles devoid of the drug, and its absorbance was subtracted from the absorbance of nanoparticles with drug. This normalized absorbance value was used in calculating the amount of drug in nanoparticles. Drug loading (DL) and encapsulation efficiency (EE) were calculated using equations 1 and 2, respectively.

DL(%w/w)=weightofandrographolide(mg)encapsulated

(1) in⁢ 1⁢mg⁢of⁢nanoparticle× 100

(2) $$\mathrm{EE}\left(\%\right)\frac{\text{Experimental}\,\text{amount}\,\text{of}\,\text{drug}\,\text{per}\,\text{mg}\,\text{nanoparticle}}{\text{Theoretical}\,\text{amount}\,\text{of}\,\text{drug}\,\text{per}\,\text{mg}\,\text{nanoparticle}}\times 100$$

#### *In vitro* Release Study

Drug release from nanoparticles was determined in (PBS, pH 7.4) with 0.2% tween 20 release buffer using a previously reported method (Toti et al., 2011). Nanoparticle suspensions (0.5 mg/mL, 2 mL) in release buffer were transferred into several tubes; the tubes were placed in a water bath shaker at 100 rpm, 37°C. At predetermined intervals (1, 2, 6, 24, 48, and 72 h), three tubes were centrifuged at 13,000 rpm for 10 min. The supernatant was analyzed for drug content via UV spectroscopy at 224 nm.

#### *In vitro* Anticancer Efficacy Studies

##### In vitro acute viability

LM2 breast cancer cells were cultured in complete MEM in an incubator at 37°C and 5% CO_2_ until they were 80% confluent. The cells were seeded in a 96-well plate (1 × 10^4^ cells in 100 μL MEM) and allowed to attach overnight. Cells were incubated with various concentrations of andrographolide solution in DMSO or equivalent concentration of nanoparticles (6.25–50 μM) for 48 h. Medium only and medium containing 50 μM of DMSO or PLGA blank nanoparticles were used as controls. At the end of the incubation period, treatments were removed, cells were washed with PBS, and 100 μL of MTS reagent (containing MTS:PMS:MEM) was added and placed in the incubator at 37°C, 5% CO_2_ for 1.5 h. Absorbance was determined at 490 nm using a microplate reader (BioTek Instruments, Inc., VT, United States). Percentage cell viability was calculated as a percentage of number of viable cells in each treatment group relative to that in the untreated control, and IC_50_ (concentration required to cause 50% reduction in the number of viable cells) was determined (Yallapu et al., 2010).

To determine the potential effects of DMSO and blank PLGA nanoparticles, the MTS assay was repeated with 20 μM of free drug, drug-loaded nanoformulation (equivalent concentration as free drug treatment), DMSO (equivalent to concentration present in the free drug), blank PLGA nanoparticles (same concentration of particles present in the nanoformulation), and medium (untreated cells). Percent cell viability was obtained for each treatment group to determine the cytotoxic effect of the DMSO solvent and blank nanoparticles on LM2 cells.

##### In vitro sustained efficacy study

The antiproliferative activity of the formulation and free drug was studied as described by Panyam et al. (Panyam and Labhasetwar, 2004). Briefly, LM2 cells were seeded in a 96-well plate (1 × 10^4^ cells) and allowed to attach overnight. Cell viability via MTS Assay was carried out as described in section “*In vitro* Acute Viability”—this represents Day 0 with no drug treatment. Cells were treated with andrographolide nanoformulation or free drug at 20 μM concentration and medium (control) for 48 h, treatments were removed and replaced with fresh medium. The medium was changed every other day thereafter, and cell viability as a function of time (representing cell proliferation) was determined via MTS Assay.

##### Cell cycle analysis

The percent cell number in different phases of cell cycle was determined using flow cytometry as described by Rajagopal et al. (2003), with slight modifications. Briefly, LM2 cells were seeded in a 6-well plate (3 × 10^5^ cells in 3 mL complete MEM) and allowed to attach overnight. The media was removed and replaced with FBS free MEM for 24 h to synchronize the cells to the same phase of the cell cycle. The cells were then incubated with free drug, andrographolide nanoformulation (equivalent to 20 μM andrographolide), or complete medium (untreated control) for 48 h. Cells were harvested by trypsinization and recovered via centrifugation at 1,000 rpm for 5 min. Cells were washed with PBS, resuspended in ice cold 70% ethanol, and incubated at 4°C for 30 min to permeabilize the cells. Cells were washed twice with flow buffer and treated with RNase (10 mg/mL) at 37°C for 15 min. The cells were then stained with propidium iodide (0.5 mg/mL) at room temperature for 2 min; cells were washed and resuspended in flow buffer. DNA content was measured using BD LSR II H4760 flow cytometer (BD Biosciences, San Jose, CA, United States), and data were analyzed using the FlowJo software.

#### Statistical Analysis

Results were reported as mean ± standard deviation (SD) or mean ± standard error of mean (SEM). Statistical differences between groups were determined using one-way analysis of variance (ANOVA) followed by Tukey’s *post hoc* test (if applicable) using the Graphpad^®^ 5 Prism software (GraphPad Software, La Jolla, CA, United States). A *p*-value <0.05 was considered significant.

## Results and Discussion

Andrographolide is a potential therapeutic agent shown to possess several beneficial pharmacological properties such as suppression of proinflammatory molecules—TNFα, inducible nitric oxide synthase (iNOS), and cyclo oxygenase 2 (COX 2); enhanced induction of immune modulator—IL-2 and induction of cell cycle arrest; and apoptosis, thereby eliciting anti-inflammatory, immunomodulatory, and anticancer activities (Pandey and Rao, 2018). The multiple anticancer mechanisms exerted by andrographolide might be useful in preventing resistance associated with conventional chemotherapeutic agents. However, its low aqueous, poor bioavailability, and short half-life results in decreased activity, hence limiting its clinical translation. To overcome these issues, andrographolide nanoformulation was developed using PLGA polymer.

### Andrographolide Nanoparticle Preparation and Characterization

The particle size of a formulation determines its *in vivo* disposition, extent of uptake by cells, and consequently its therapeutic efficacy. It is generally accepted that for *in vivo* applications, smaller particle size is preferred. Particles in the 1–3 nm are prone to clearance by renal filtration, while large particles are rapidly cleared by the reticuloendothelial system, thereby reducing their circulation time (Yin Win and Feng, 2005; Sadat et al., 2016). Furthermore, particles greater than 200 nm when administered intravascularly may cause embolization (Hickey et al., 2015). Nanoparticles in the 50–200 nm size have demonstrated the highest percentage of cellular uptake (Yin Win and Feng, 2005). However, nanoparticles that are less than 50 nm suffer from poor payload capacity (Jain and Thareja, 2019). Therefore, our desired particle size was 50 to 200 nm.

Polydispersity Index is a measure of size distribution within a given sample (Danaei et al., 2018); it ranges from 0.0 (a perfect homogenously dispersed size population) to 1.0 (a heterogeneously dispersed system with multiple size populations). Formulations with wide range of particle distribution result in variations in DL, which will in turn lead to variability in drug release, bioavailability, and eventually efficacy (Betala et al., 2018). Formulations with PI ≤ 0.20 are generally acceptable for a polymer-based nanoformulation (Clarke, 2013; Danaei et al., 2018).

Zeta potential predicts the stability of a nano dispersion; higher absolute values (that is, either positive or negative) of zeta potential result in better suspension stability due to the presence of strong repulsive forces that prevent aggregation of particles (Sawant and Dodiya, 2008; Kedar et al., 2010). However, high surface charge on particles has been shown to result in increased macrophage uptake, resulting in increased clearance, reduced bioavailability and therapeutic efficacy (Honary and Zahir, 2013; Sadat et al., 2016). A formulation with decreased absolute value of surface charge and near zero value may have higher circulation time and higher accumulation in the tumor. For example, a previous study suggested that a formulation with particle size of about 150 nm and a slightly negative surface charge tend to accumulate more within tumor (Honary and Zahir, 2013; Sadat et al., 2016).

High DL and EE enables a reduction in the total amount of the formulation (and by extension, the formulation excipients) that needs to be administered for a given dose of the drug, thus preventing excipient-associated toxicity.

In the current study, we investigated the effect of various formulation parameters with the objective of optimizing the key nanoparticle properties discussed above.

#### Effect of PLGA Lactide:Glycolide Ratio

The effect of varying the lactide to glycolide ratio on various nanoparticle properties is shown in Table 2. In general, no correlation was observed between lactide to glycolide ratio and any of the physical properties. The average particle size varied from 194 to 209 nm, while the PI varied from 0.08 to 0.20 and the zeta potential from −13.5 to −23.5 mV. The DL of the formulations was in the 1.0–1.5%w/w range, with EE of 7.5 –11.5%. The 50:50 PLGA was chosen for further studies because that polymer consistently resulted in high DL compared to other polymers.

**TABLE 2:** Physicochemical properties and drug loading of andrographolide formulation with different lactide to glycolide ratio.

**Formulation code**	**Particle size (nm)**	**Polydispersity index (PI)**	**Zeta potential (mV)**	**Drug loading (%)**	**Encapsulation efficiency (%)**
A1	2093	0.200.01	−23.53.8	1.50.4	11.52.9
A2	1977	0.140.03*	−14.40.1*	1.00.2	7.51.5
A3	2003	0.090.01***	−13.52.5*	1.10.3	9.12.8
A4	1942*	0.100.01***	−17.25.6	1.30.2	9.81.7
A5	2026	0.080.02***	−16.21.8	1.20.1	8.71.0

Results are expressed as mean ± SD (*n* = 3). *signifies *p* < 0.05, ***signifies *p* < 0.001 significant differences with respect to A1. A1, 50:50 PLGA; A2, 65:35 PLGA; A3, 75:25 PLGA; A4, 85:15 PLGA; A5, 100 PLA.

#### Effect of Organic Solvents

The effect of organic solvent used on nanoparticle properties is described in Table 3. Andrographolide nanoparticles made with 50:50 PLGA polymer and different organic solvents produced formulations having mean particle size in the range of 112 to 240 nm, PI in the range of 0.10 to 0.20, zeta potential of −10.6 to −23.5 mV, DL of 1.5 to 2.3%w/w, and EE of 11.5 to 18.2%.

**TABLE 3:** Physicochemical properties and drug loading of andrographolide nanoformulation with different solvent.

**Formulation code**	**Particle size (nm)**	**Polydispersity index (PI)**	**Zeta potential (mV)**	**Drug loading (%)**	**Encapsulation efficiency (%)**
B1	2093	0.200.01	−23.53.8	1.50.4	11.52.9
B2	2407***	0.130.07	−17.33.5	1.70.3	12.82.0
B3	1126***	0.200.02	−11.40.9*	2.30.3	18.22.1
B4	2198	0.100.01*	−10.64.7*	2.30.8	17.86.2

Results are expressed as mean ± SD (*n* = 3). *signifies *p* < 0.05, ***signifies *p* < 0.001 significant differences with respect to B1 formulation. B1, chloroform; B2, dichloromethane; B3, ethyl acetate; B4, acetone.

The chloroform (B1) and acetone (B4) formulations had similar particle size while the dichloromethane (B2) and ethyl acetate (B3) formulations had the largest and smallest particle size, respectively. A similar observation of reduced particle size with ethyl acetate organic solvent was demonstrated by Vineeth et al. (Vineeth et al., 2014); this is attributed to the low interfacial tension of ethyl acetate, which allows for the formation of a stable primary emulsion and consequently formation of smaller nanoparticles (Vineeth et al., 2014). All of these formulations except the one that utilized acetone had similar heterogeneity in size distribution; the acetone formulation had a lesser variation in size uniformity than the other three formulations. The chloroform and dichloromethane formulations (having similar values) had higher absolute charge but lower DL and EE than the ethyl acetate and acetone formulations (possessing similar charge, DL, and EE). The differences in the DL for the various formulations could have resulted in the differences in their zeta potential. The higher DL and EE observed in ethyl acetate and acetone formulations could be attributed to the properties of the solvents. Ethyl acetate and acetone have higher aqueous solubility than chloroform and dichloromethane; this could keep the drug soluble in the emulsion during the encapsulation process, allowing more of the drug to be entrapped in the polymer (Pauli et al., 2019). The ethyl acetate and acetone formulations therefore represent the preferred formulations with respect to surface charge, DL, and EE. The ethyl acetate formulation was chosen for further evaluation because of the lower particle size, higher DL, and EE.

#### Effect of PLGA Molecular Weight

Andrographolide nanoformulations prepared using ethyl acetate organic solvent and 50:50 PLGA polymer of different molecular weights (as measured through polymer inherent viscosities) had a mean particle size in the range of 107–143 nm, PI of 0.10–0.20, zeta potential of −8.1 to −11.4 mV, DL of 1.1–2.3%w/w, and EE of 8.4–18.2% as shown in Table 4.

**TABLE 4:** Physicochemical properties and drug loading of andrographolide formulation of different PLGA molecular weights.

**Formulation code**	**Particle size (nm)**	**Polydispersity index–PI**	**Zeta potential (mV)**	**Drug loading (%)**	**Encapsulation efficiency (%)**
C1	1073	0.200.03	−10.40.9	1.20.1***	9.00.6***
C2	1126	0.200.02	−11.40.9	2.30.3	18.22.1
C3	1394***	0.100.05*	−8.10.8*	1.10.2***	8.41.4***
C4	1436***	0.120.04	−10.30.1	1.50.1**	11.60.9**

Results are expressed as mean ± SD (*n* = 3). *signifies *p* < 0.05, **signifies *p* < 0.01, ***signifies *p* < 0.001 significant differences with respect to C2 formulation. C1, 6.7–31.3 kDa; C2, 31.3–57.6 kDa; C3, 57.6–91.6 kDa; C4, 91.6–111.5 kDa.

The 6.7–31.3 kDa (C1) and 31.3–57.6 kDa (C2) formulations had similar smaller size than those of 57.6–91.6 kDa (C3) and 91.6–111.5 kDa (C4) formulations. This suggests that higher molecular weight of the polymer results in larger particle size. The increase in size associated with increased molecular weight can be attributed to the formation of a more viscous solution, which provides resistance to particle size breakdown, and thus more energy is required to achieve smaller particle size. All the formulations had similar size distribution and charge except for the 57.6–91.6 kDa formulation, which demonstrated lesser size variation and lower absolute surface charge value. However, this formulation had the lowest DL. The highest DL and EE was observed in the 31.3–57.6 kDa polymer formulation. This polymer was chosen for further studies because of its small particle size, comparable size heterogeneity, and surface charge to the other formulations, highest DL, and EE.

#### Effect of Drug–Polymer Ratio

Andrographolide formulations with ethyl acetate organic solvent, 50:50 PLGA polymer (molecular weight 31.3–57.6 kDa) and having different drug–polymer ratios had a mean particle size in the range of 112 to 148 nm, PI of 0.18 to 0.21, zeta potential of −8.1 to −11.4 mV, DL of <1.0 to 2.3%w/w, and EE of <9.8 to 23.2% as shown in Table 5.

**TABLE 5:** Physicochemical properties and drug loading of andrographolide nanoformulation with different drug-polymer ratio.

**Formulation code**	**Particle size (nm)**	**Polydispersity index–PI**	**Zeta potential (mV)**	**Drug loading (%)**	**Encapsulation efficiency (%)**
D1	1444***	0.180.03	−11.31.2	Unquantifiable	Unquantifiable
D2	1485***	0.200.02	−8.21.2	1.00.0***	13.90.4
D3	1332**	0.200.03	−10.12.0	2.20.3	23.23.7
D4	1126	0.200.02	−11.410	2.30.3	18.22.1
D5	1309*	0.210.04	−8.12.3	1.80.3	9.81.8**

Results are expressed as mean ± SD (*n* = 3). *signifies *p* < 0.05, **signifies *p* < 0.01, ***signifies *p* < 0.001 significant differences with respect to D4 formulation. D1, 1:20; D2, 1:12; D3, 1:8.5; D4, 1:6; D5, 1:4.

The 1:20 (D1) and 1:12 (D2) drug–polymer ratio formulations were characterized by larger particle size than the 1:8.5 (D3) and 1:4 (D5) formulations, while the 1:6 (D4) formulation had the least particle size. All the formulations comprised particles with similar size distribution and surface charge. The DL of the 1:20 formulation could not be determined because the drug–polymer ratio was so low that the amount of drug encapsulated could not be detected or quantified accurately. Increase in drug-polymer ratio led to an increase in the amount of drug encapsulated until the 1:6 drug–polymer ratio; a further increase did not yield an increase in DL as observed in the 1:4 formulation. An initial increase in EE was observed with higher drug-polymer ratio, however, a further increase to 1:6 drug-polymer ratio led to a decrease in the EE even though the amount of drug encapsulated is comparable to that of 1:8.5 formulation. This implies that an increase in the quantity of drug utilized in the formulation beyond the 1:8.5 ratio will be a waste of the drug material given that there seems to be no appreciable improvement in DL. The 1:8.5 ratio was therefore chosen for further studies due to its high DL and EE, size distribution and surface charge comparable to other formulations, and relatively small particle size (even though the size was greater than the 1:6 formulation—133 nm vs 112 nm, respectively, it was still within the desired 50–200 nm range).

#### Effect of Surfactant Concentration

Andrographolide formulations with ethyl acetate organic solvent, 50:50 PLGA polymer (molecular weight 31.3–57.6 kDa), 1:8.5 drug–polymer ratio, and different PVA surfactant concentrations had a mean particle size in the range of 114–163 nm, PI of 0.16–0.20, zeta potential of −10.1 to −14.1 mV, DL of 1.2–2.8%w/w, and EE of 12.6–27.3% as shown in Table 6.

**TABLE 6:** Physicochemical properties and drug loading of andrographolide nanoformulation with different PVA concentration.

**Formulation code**	**Particle size (nm)**	**Polydispersity index–PI**	**Zeta potential (mV)**	**Drug loading (%)**	**Encapsulation efficiency (%)**
E1	1631***	0.170.02	−14.11.1	2.50.2	24.31.7
E2	1144***	0.170.04	−11.61.3	2.80.4*	27.33.6
E3	1332	0.200.03	−10.12.0	2.20.3	23.23.7
E4	1371	0.200.03	−12.60.3	1.20.1**	12.60.5**
E5	1291	0.160.07	−13.53.5	1.30.1**	13.40.9**

Results are expressed as mean ± S.D (*n* = 3). *signifies *p* < 0.05, **signifies *p* < 0.01, ***signifies *p* < 0.001 significant differences with respect to E3 formulation. E1, PVA 1% w/v, E2, PVA 2% w/v, E3, PVA 2.5% w/v, E4, PVA 3% w/v, E5, PVA 4% w/v.

The 1% (E1) and 2% w/v (E2) PVA formulations had the largest and smallest mean particle size, respectively, and there was no relationship between the size and surfactant concentration. The PVA concentration did not affect the size distribution and surface charge of the particles as similar PI and zeta potential were obtained in all the formulations. An increase in the surfactant concentration resulted in an increase in the amount of drug encapsulated up to the 2% concentration; a further increase caused a reduction in DL and EE as observed for the 2.5% (E3), 3% (E4), and 4% w/v (E5) PVA concentrations. This can be attributed to the ability of the surfactant to improve the solubility of poorly water-soluble substances in aqueous medium (Vinarov et al., 2018); an increase in surfactant concentration will lead to more andrographolide present in the aqueous phase of the emulsion being lost during washing, resulting in lower DL and EE.

The 2% w/v PVA formulation was found to be the most suitable because it had the smallest particle size but with size distribution and surface charge comparable to other formulations and highest drug content and EE.

### *In vitro* Release Studies of Andrographolide Nanoformulation

**FIGURE 1:**
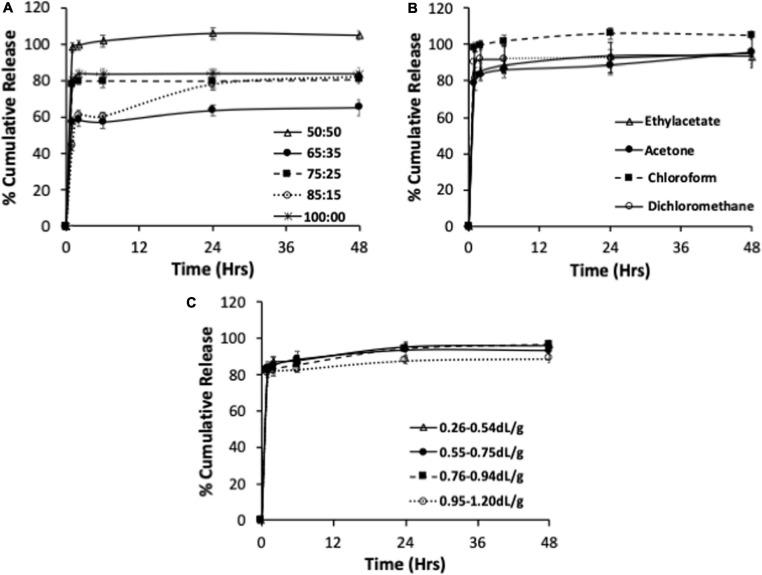
*In vitro* release of andrographolide nanoformulations prepared using different lactide to glycolide ratios **(A)**, organic solvent **(B)**, and PLGA molecular weights **(C)**. Data expressed as mean ± S.D (*n* = 3).

The release profiles of andrographolide from formulations having different lactide to glycolide ratios, organic solvent, and PLGA molecular weights are represented in Figures 1A–C.

Drug release from polymeric dispersions can occur through several mechanisms including via polymer degradation, desorption from the particle surface followed by diffusion from the bulk, or a combination of these mechanisms (Alhakamy and Md, 2019). PLGA is known to undergo bulk erosion and release of hydrophobic drugs from PLGA matrices occurs through a combination of drug diffusion (dominant during the early phases) and polymer degradation (more dominant during terminal phase) (Makadia and Siegel, 2011). The formulations prepared using polymers of varying lactide to glycolide ratios released their total andrographolide content in 2–48 h (Figure 1A). The 75:25, 85:15 PLGA and 100:0 PLA resulted in similar amount of andrographolide release, and this was lower than the 50:50 formulation. The slowest drug release was observed for the 65:35 PLGA formulation. The mechanism(s) underlying this observation is unclear. One possibility is that reduction in the total drug release in the 65:35, 75:25, 85:15, and 100% PLA when compared to the 50:50 formulation might be due to the increase in the hydrophobic content of the polymer conferred by higher lactide content. This might have led to increased affinity of the drug to the polymer, resulting in slower drug release (Park, 1995; Lee et al., 2002). However, both 75:25 PLGA and 100 PLA formulations released their total drug content within 2 h. In contrast, the 65:35 and 85:15 formulations demonstrated a gradual release over 24 and 48 h, with an initial burst release of 57 and 60%, respectively, in 2 h. Thus, the release profile did not directly correlate with the lactide content or the hydrophobicity of the polymer. Differences in particle size and DL for the different formulations could have also contributed to the differences in the observed drug release profiles.

All the formulations prepared using different organic solvents released their andrographolide content between 24 and 48 h (Figure 1B). The chloroform and ethyl acetate nanoformulations released their drug content in 24 h while the dichloromethane and acetone formulations release their drug over 48 h. All the formulations resulted in a rapid initial burst release of at least 84% within 2 h. The initial rapid release was slightly lower in ethyl acetate and acetone formulations than for dichloromethane and chloroform formulations. The 50:50 PLGA polymer appeared to result in rapid release of the drug content irrespective of the solvent used in the fabrication of the formulation.

The andrographolide nanoformulations obtained from 50:50 PLGA polymer of different molecular weights released their drug content in 24 h (6.7–31.3, 31.3–57.6, and 91.6–111.5 kDa polymeric formulation) to 48 h (57.6–91.6 kDa polymeric formulation) (Figure 1C). The andrographolide formulation prepared with a high molecular weight polymer, 91.6–111.5 kDa, demonstrated a slight reduction in the total andrographolide release (88%) when compared with the 6.7–31.3, 31.3–57.6, and 57.6–91,6 kDa formulations that resulted in similar drug release (96, 93, and 97% andrographolide release, respectively). All the 50:50 PLGA inherent viscosity formulations showed a rapid initial release of at least 82% of its drug content within 2 h.

The burst release observed in the formulations can be attributed to both the presence of surface-associated drug and the large surface area of PLGA nanoparticles, which allows for rapid drug diffusion. These physicochemical properties are influenced by factors such as molecular weight of the polymer, polymer concentration and hydrophilicity of the polymer (Mohammadi-Samani and Taghipour, 2015). Therefore, further optimization of the polymer properties may result in a formulation with less burst effect.

**FIGURE 2:**
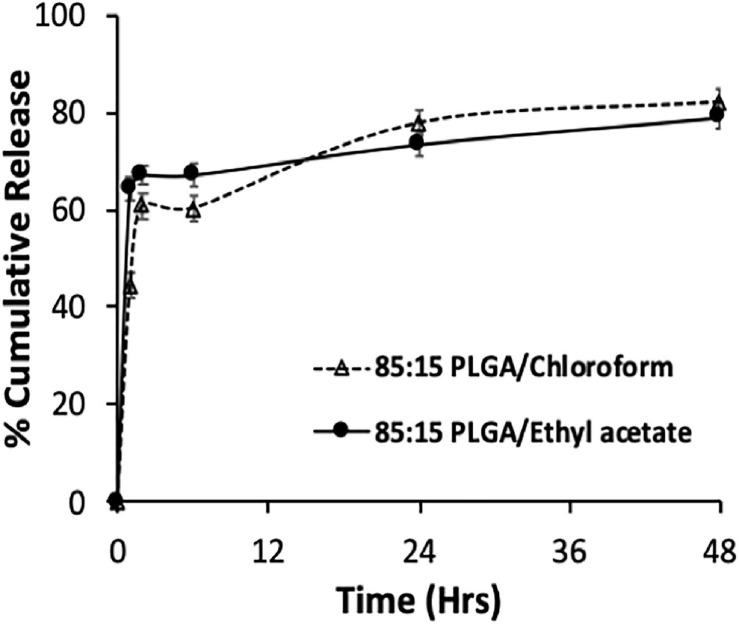
*In vitro* release of andrographolide nanodispersion prepared using 85:15 PLGA polymer and chloroform or ethyl acetate as the organic solvent. Data expressed as mean ± SD (*n* = 3).

Considering that most of the drug was released in few hours in most formulations, the 85:15 PLGA polymer was chosen for the preparation of nanoparticle and subsequent evaluation of its antiproliferative activity on breast cancer cells because it exhibited sustained release potential and high total drug content release (∼80% release over 48 h). The andrographolide nanoformulation was made with ethyl acetate as the organic solvent (since it resulted in formulations with reduced particle size and increased DL), 85:15 PLGA polymer, drug-polymer ratio of 1:8.5 and 2% PVA; physicochemical properties, DL, EE, and release of this formulation are shown in Table 7 and Figure 2.

**TABLE 7:** Physicochemical properties and drug loading of optimized andrographolide nanoformulation.

**Formulation code**	**Particle size (nm)**	**Polydispersity index–PI**	**Zeta potential (mV)**	**Drug loading (%)**	**Encapsulation efficiency (%)**
Ethyl acetate	1354***	0.220.00***	−11.72.4	2.60.6*	19.14.1***
Chloroform	1942	0.100.01	−17.25.6	1.30.2	9.71.7

Results are expressed as mean ± SD (*n* = 3). *signifies *p* < 0.05, ***signifies *p* < 0.001 significant differences with respect to 85:15 chloroform formulation.

The 85:15 ethyl acetate andrographolide formulation had smaller particle size, increased DL, higher EE, comparable zeta potential, and a more heterogenous particle size distribution when compared with the chloroform formulation. The formulations fabricated using ethyl acetate and chloroform released a total of 79 and 82%, respectively, within 48 h. Both formulations exhibited a similar release pattern, however, more andrographolide was released from the ethyl acetate formulation initially (64 vs 44% at 1 h; 67 vs 60% at 2–6 h for ethyl acetate and chloroform, respectively). This can be attributed to the size of the formulation—the smaller the size, the larger the surface area and the faster the rate of drug release (Rizvi and Saleh, 2018). Based on these desirable properties of smaller size, increased DL, and more sustained *in vitro* release profile, the formulation prepared using the 85:15 PLGA polymer and ethyl acetate as the organic solvent was chosen as the optimized formulation for cell culture studies.

### Surface Morphology of Optimized Andrographolide Formulation

**FIGURE 3:**
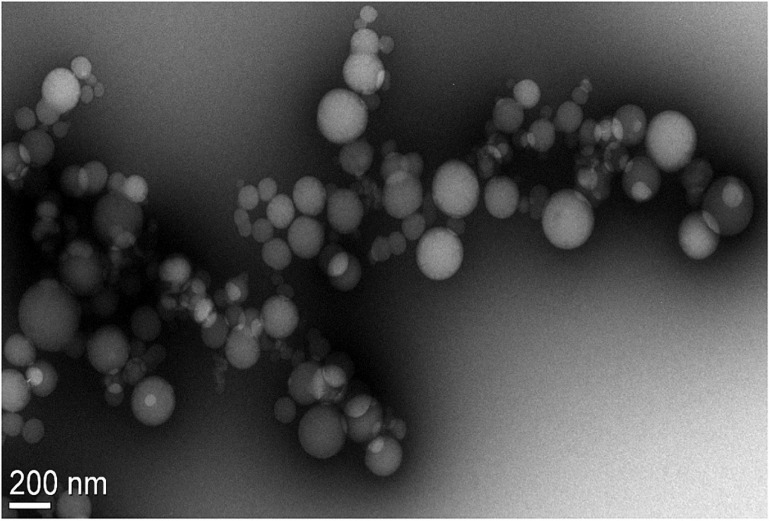
TEM image of Andro 85:15 EA formulation showing discrete spherical particles ranging from 63 to 206 nm in diameter measured using the Gatan^®^ Digital Micrograph software (Pleasanton, CA, United States).

A TEM image of the 85:15 PLGA ethyl acetate formulation showed discrete, spherical particles with sizes ranging from 63 to 206 nm (Figure 3). This appeared to correlate well with the particle size and size distribution determined using DLS (Table 7).

### *In vitro* Anticancer Efficacy Studies With the Optimized Andrographolide Formulation

#### *In vitro* Acute Viability

**FIGURE 4:**
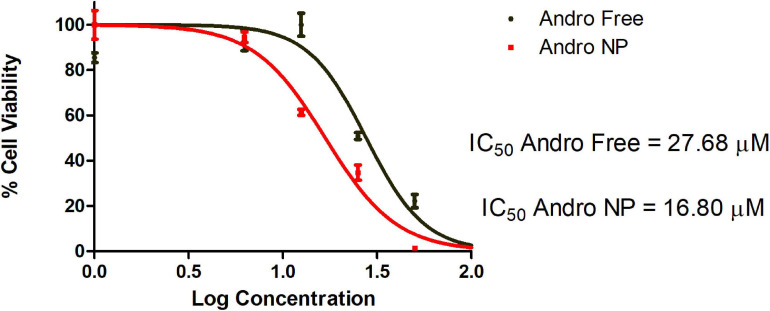
Dose-response curve of andrographolide free drug and nanoformulation on LM2 cells following 48 h treatment. Andro Free, andrographolide free drug, Andro NP, andrographolide nanoformulation.

Initial studies evaluated the acute effect of the andrographolide free drug and the nanoformulation on cell viability over 48 h. This study showed that the nanoformulation was better than the free drug (IC_50_ of 27.68 μM for free drug vs 16.80 μM for nanoformulation) as shown in Figure 4.

**FIGURE 5:**
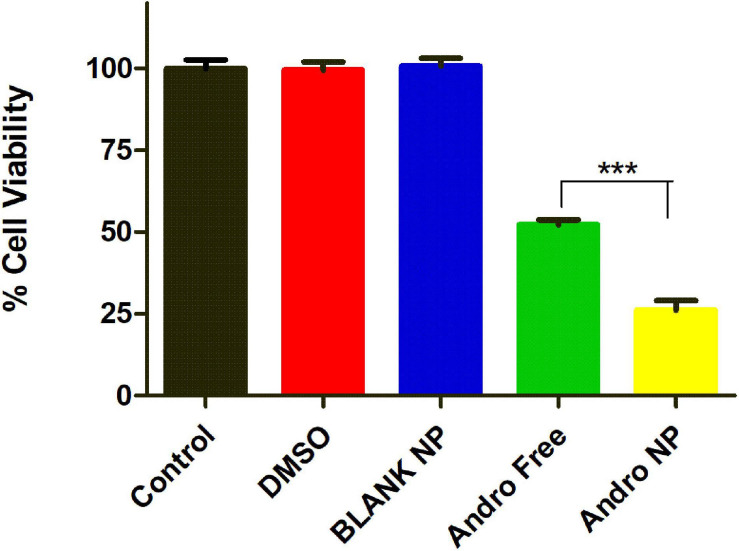
Cell viability of DMSO, blank NP, andrographolide free, and nanoformulation treated LM2 cells showing no cytotoxic effect for DMSO and PLGA nanoparticle at 20 μM after 48 h treatment. Blank NP, blank PLGA nanoformulation, Andro Free, andrographolide free drug, Andro NP, andrographolide nanoformulation.

To determine the effect of blank particles and solvent (DMSO), the LM2 cells were treated with the same concentration of DMSO and blank PLGA nanoparticles present in the free drug and nanoformulation treated group. After 48 h, the DMSO and blank PLGA nanoparticle treated cells showed similar viability (99.5% and 100.8%, respectively) as the untreated (100%) group (Figure 5). This demonstrates that the DMSO solvent or the PLGA polymer did not have cytotoxic effects on the LM2 cells at the concentration utilized. As in the previous study, nanoformulation was more cytotoxic than the equivalent concentration of the free drug. Untreated cells were used as controls in further experiments since DMSO and blank PLGA nanoformulation demonstrated no cytotoxic activity.

#### *In vitro* Sustained Efficacy

**FIGURE 6:**
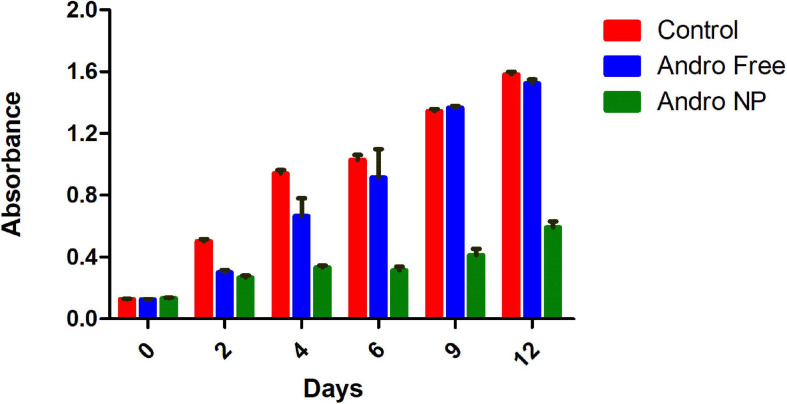
Cell viability of untreated LM2 cells, andrographolide free and nanoformulation post treatment removal showing prolonged cytotoxic effect of andrographolide nanoformulation. Andro Free, andrographolide free drug, Andro NP, andrographolide nanoformulation.

We then evaluated the effect of nanoformulation on cell viability over 12 days. The cell viability of andrographolide free drug, andrographolide nanoformulation and untreated cells (control) at various time points following treatment removal is shown in Figure 6.

Prior to treatment (day 0), the cells seeded showed similar absorbance, indicating similar number of cells present in the different groups. Upon treatment removal, the free drug treatment group showed a transient cytotoxic effect for up to 6 days post treatment. This effect was lost after 6 days, as demonstrated by the presence of similar number of viable cells as in the untreated group. In contrast, the cells treated with andrographolide nanoformulation maintained lower cell numbers upon treatment removal until the 12th day of the study. Thus, the nanoformulation demonstrated a sustained cytotoxic effect. This is in line with the findings of Panyam and Labhasetwar (2004). The sustained inhibition of cell proliferation observed in PLGA nanoformulation in that study was attributed to the sustained intracellular drug levels as opposed to that with the free drug in which intracellular drug levels decreased drastically upon removal of the treatment (Panyam et al., 2002; Panyam and Labhasetwar, 2004).

#### Cell Cycle Analysis

**FIGURE 7:**
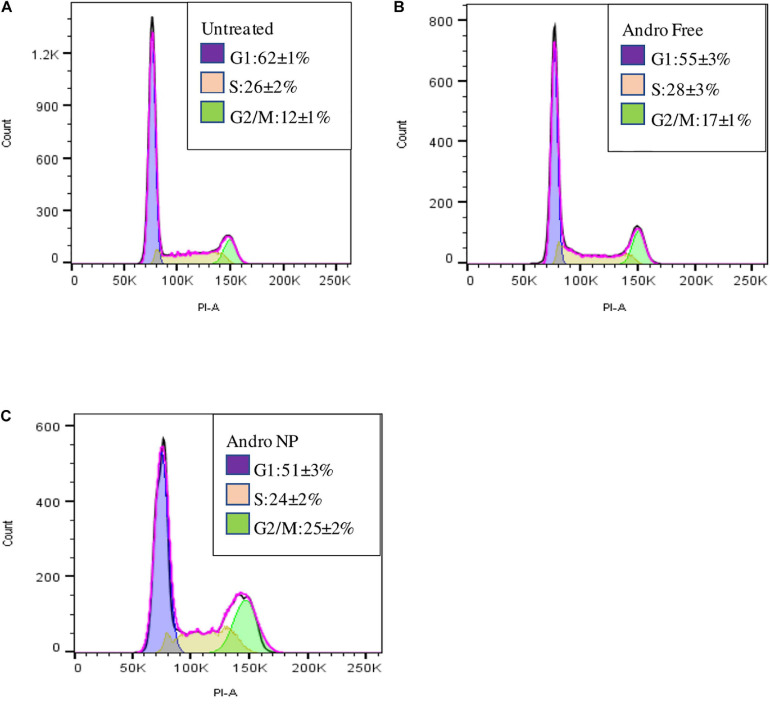
Cell cycle of LM2 cells showing the proportion of cells in the cell cycle phases for untreated cells **(A)**, free andrographolide treated cells **(B)**, and nanoformulation treated cells **(C)**.

Cell cycle analysis demonstrated accumulation of cells in the G2/M phase in both andrographolide free drug and nanoformulation compared to that with untreated cells as shown in Figures 7A–C.

The andrographolide free drug and nanoformulation treatments resulted in a decrease in the number of cells in the G1 phase with an increase in the G2/M phase when compared to that with the untreated cells. This is in line with a study carried out by Banerjee et al., in which andrographolide elicited cell cycle arrest in the G2/M phase in MDA-MB-231 cells—the parent cell line of LM2 cells used in this study (Banerjee et al., 2016). A higher number of cells were in the G2/M phase for the nanoformulation treated group than in the group treated with the free drug, demonstrating improved therapeutic effect with the nanoformulation.

## Conclusion

A polymeric nanoformulation of andrographolide was developed, and the effect of different formulation parameters on physicochemical properties and release profile was determined to obtain a formulation with desirable properties. Encapsulation of andrographolide in nanoparticles of approximately 100–150 nm size was achieved using ethyl acetate as the organic solvent. Nanoparticles formulated using 85:15 lactide to glycolide ratio PLGA polymer, drug–polymer ratio of 1:8.5, 2% PVA, and ethyl acetate as the organic solvent were identified as the optimized formulation for andrographolide. This formulation demonstrated enhanced and sustained inhibition of proliferation of triple negative LM2 breast cancer cells when compared to the free drug. This formulation can serve as a template for further development of andrographolide as a potential anticancer agent for clinical use.

## Data Availability Statement

The raw data supporting the conclusions of this article will be made available by the authors, without undue reservation.

## Author Contributions

BO, CA, OO, and JP conceptualized the study. BO and JP developed the methods for the study. BO carried out the experiments, analyzed the data obtained, and wrote the manuscript draft in collaboration with JP. CA, OO, CI, and JP supervised the research and reviewed the manuscript. JP acquired the funds for the study. All authors have read and agreed to the published version of the manuscript.

## Conflict of Interest

The authors declare that the research was conducted in the absence of any commercial or financial relationships that could be construed as a potential conflict of interest.
